# BET inhibitors: Betting on improved outcomes in uterine serous carcinoma

**DOI:** 10.18632/oncotarget.26245

**Published:** 2018-10-26

**Authors:** Burak Zeybek, Salvatore Lopez, Alessandro D. Santin

**Affiliations:** Department of Obstetrics, Gynecology, and Reproductive Sciences, Yale University School of Medicine, New Haven, CT, USA

**Keywords:** endometrial cancer, uterine serous carcinoma, bromodomain and extraterminal motif proteins, GS-626510, GS-5829

During the last decade, cancer research has significantly evolved into precision medicine and targeted therapies are now part of the many standard treatment regimens in various cancers. As recent cancer genome projects highlighted the importance of epigenetic alterations in cancer development, this area has become a growing focus of interest; thus several drug classes are now being studied that target various components of epigenetic network. One of these epigenetic targets is the bromodomain and extraterminal motif (BET) proteins, which play key roles in gene transcription and cell cycle regulation [1]. The BET family includes four proteins; BRD2, BRD3, BRD4, and bromodomain testis-specific protein (BRDT), all of which consists of two N-terminal bromodomains. These bromodomains are a group of structurally similar proteins, which serve as epigenetic ‘readers’ by recognizing and binding acetylated lysine residues on histone tails. The first two BET inhibitors, I-BET and JQ1, were described in 2010; I-BET was found to selectively down-regulate the expression of proinflammatory genes and transcription factors [2], and JQ1 was found to have significant activity against NUT midline carcinoma [3]. The mechanism of the latter was shown to be displacement of BRD4 from chromatin that led to G1 cell cycle arrest and apoptosis. Perhaps, the most compelling observation of BET inhibitors arose from the study by Jang et al, in which transfection of BRD4 increased the activity of cellular promoters, c-Myc and c-Jun [4]. Also positive transcription elongation factor b (P-TEFb), which is involved in most of RNA polymerase II – dependent transcriptions, was not localized to the c-Myc locus during G1 in BRD4 knockdown cells, indicating that BRD4 plays a critical role for c-Myc transcription. This interaction was later demonstrated in various cancer cell lines, such as leukemia and medulloblastoma [5, 6].

The incidence and mortality rates of endometrial cancer (EC) are globally increasing, and, if current trends continue, the incidence of EC will double by 2030 in the United States [7]. However, there have been no new drugs approved for the treatment of EC in decades. The majority of deaths are caused by highly aggressive histologic types such us uterine serous carcinoma (USC), a variant accounting for less than 10% of all endometrial tumors [8]. Recent whole-exome sequencing (WES) studies from our laboratory demonstrated high genomic instability and gain-of-function mutations in c-Myc in USC, suggesting c-Myc as a potential target in this highly aggressive variant of EC [9]. In the light of these findings, we recently investigated the activity of two novel BET inhibitors, GS-5829 and GS-626510, against primary USC cultures and USC xenografts. *In vitro* experiments demonstrated high activity of these agents against USC cell lines with high c-Myc expression by causing a dose-dependent decrease in the phosphorylated levels of c-Myc and a dose-dependent increase in caspase activation. *In vivo* results were also similar, in which both GS-5829 and GS-626510 exhibited a significantly slower rate of tumor growth, compared with vehicle control as well as mice undergoing treatment with JQ1 (*p* < 0.05). Importantly, the drug activity was not affected by the presence or absence of gain of function mutations in the PIK3CA gene, which was previously shown to be related with resistance to trastuzumab treatment in USC [10]. No evidence of acute or chronic toxicity was observed in the treated animals. GS-5829 is currently being evaluated in multiple phase I–II clinical trials in solid tumors and lymphomas (ClinicalTrials.gov identifier: NCT02392611, NCT02607228, NCT02983604), while GS-626510, due to its shorter half-life, has so far been assessed only in preclinical studies.

The relationship between cMyc regulation and BET inhibition is complex due to the fact that BET proteins have many roles beyond cancer and the effects of Myc suppression are not uniform across tumor types. However, our results are promising and deserve further investigation. Myc gain-of-function may represent a novel target in USC and treatment with BET inhibitors used alone or in combination with other targeted agents might be a potential option in patients with advanced/recurrent USC unresponsive to chemotherapy.
